# Obstetric and perinatal effects of active and/or passive smoking during pregnancy

**DOI:** 10.1590/S1516-31802004000300004

**Published:** 2004-05-06

**Authors:** Mary Uchiyama Nakamura, Sandra Maria Alexandre, Jorge Francisco Kuhn dos Santos, Eduardo de Souza, Nelson Sass, Anna Paula Auritscher Beck, Evelyn Trayna, Carla Maria de Araújo Andrade, Teresa Barroso, Luiz Kulay

**Keywords:** Smoking, Pregnancy, Passive smoking, Birth weight, Spontaneous abortion, Cigarro, Tabagismo, Gravidez, Fumar passivo, Peso ao nascer, Aborto espontâneo

## Abstract

**CONTEXT:**

Cigarette smoke, whether inhaled voluntarily or not, causes damage to the mother-infant pair. The antenatal period may present the best opportunity for performing effective anti-smoking campaigns.

**OBJECTIVE:**

To study the obstetric and perinatal effects of smoking on pregnancy and the infant.

**TYPE OF STUDY:**

Prospective study, interviewing pregnant women who were randomly selected at the maternity hospital as they were being discharged after giving birth.

**SETTING:**

Hospital Municipal Vereador José Storópolli, São Paulo, Brazil.

**METHODS:**

758 patients were interviewed regarding smoke inhalation before being discharged from the maternity hospital. The groups were formed by 42 active smokers, 272 passive smokers, 108 who inhaled smoke both actively and passively, and 336 non-smokers. The groups were compared regarding age, parity, school education, incidence of spontaneous abortion, rate of caesarian births, average gestational age at birth, rate of low birth weight and adequacy of weight in relation to the gestational age of newborn infants. For all variables we considered p < 0.05 as statistically significant.

**RESULTS:**

There was a high rate (55.7%) of pregnant smokers, including 5.5% active, 35.9% passive and 14.3% active-passive smokers. Active and active-passive smokers were older and had higher parity. Active smokers had lower education levels and higher rates of previous spontaneous abortion. The weights of newborn babies were lower for smoking mothers.

**DISCUSSION:**

The study was performed among patients that were mostly of low economic, social and cultural levels, thus possibly explaining the high incidence of smokers. Worse still was that 35.9% of the non-smokers were actually passive smokers. These rates we report were similar to those from the literature. The typical receptiveness of teenage girls to unrestricted advertising in the media contributes towards an early start to acquiring the habit of smoking, including during pregnancy in our country. We emphasize the difficulties in quantifying exposure to cigarettes even among active smokers.

**CONCLUSIONS:**

Cigarette smoke, whether inhaled voluntarily or not, has an unfavorable effect on the mother-infant pair.

## RESUMO

**CONTEXTO:**

Objetivamos neste estudo avaliar as repercussões obstétricas e perinatais do tabagismo. A fumaça do cigarro inalada de forma voluntária ou não traz prejuízos sobre o binômio materno-fetal. O período pré-natal pode ser a melhor oportunidade para a realização de campanhas antitabagistas mais efetivas.

**OBJETIVO:**

Estudar as repercussões do tabagismo na gravidez e no recém-nascido.

**TIPO DE ESTUDO:**

Prospectivo, por meio de entrevistas realizadas entre as pacientes inter-nadas na maternidade, escolhidas aleatoriamente após a resolução da gravidez.

**LOCAL:**

Hospital Municipal Vereador José Storópolli, São Paulo, Brasil.

**MÉTODOS:**

758 Pacientes foram entrevistadas quanto ao tabagismo previamente à alta da maternidade. Os grupos estudados foram compostos de 42 fumantes ativas, 272 fumantes passivas, 108 que inalaram ativa e passivamente, e de 336 mulheres não-fumantes. Os grupos foram comparados entre si quanto a idade, paridade, grau de escolaridade, incidência de abortamento, taxa de cesariana, média da idade gestacional no parto, a freqüência de baixo peso e adequação peso/idade gestacional dos recém nascidos. Para todas as variáveis, consideramos p < 0,05 como estatisticamente significante.

**RESULTADOS:**

Observamos elevada incidência de tabagismo na gravidez (55,7%) incluindo fumantes ativas (5,5%), passivas (35,9%) e ativas-passivas (14,3%). As fumantes ativas e ativas-passivas mostraram-se com idade e paridade superiores, enquanto as ativas apresentavam menor grau de instrução e maior incidência de abortamentos prévios. Os neonatos de mães tabagistas tiveram menor peso.

**DISCUSSÃO:**

O estudo foi realizado principal-mente com pacientes de baixo nível sócioeconomico-cultural, possivelmente explicando a elevada incidência de tabagistas. Ressaltamos que 35,9% das não-fumantes eram, na realidade, fumantes passivas. Estes resultados foram similares àqueles da literatura. O mesmo pode ser dito em relação a ocorrência de aborto e recém-nascidos de baixo peso. A nicotina não filtrada está presente na fumaça de cigarro ambiental, cujo policiamento governamental da poluição se mostra inefetivo. Além disso, atitude típica das jovens adolescentes de permissividade e aces-so à livre propaganda do cigarro na mídia contribuem para um início precoce de aquisição do vício durante a gravidez no nossos País. Enfatizamos as dificuldades em quantificar a exposição ao cigarro mesmo em fumantes ativas, pior ainda para as passivas.

**CONCLUSÕES:**

A fumaça do cigarro inalada de forma voluntária ou não repercute desfavoravelmente sobre o binômio materno-fetal.

## INTRODUCTION

Smoking is the cause of the highest rates of preventable diseases and premature death in the history of the human race. It is now considered that there is a worldwide epidemic of smoking-related diseases, since these are responsible for 4 million deaths a year (according to the World Health Organization, 1998^1^), corresponding to 10 thousand deaths a day or one every 8 seconds. Such diseases have high social and economic costs.

In Brazil, the National Cancer Institute (Inca) estimates that a third of Brazilian adults smoke and that approximately 11.2 million of them are women. 90 % of them become addicted at an early age, between five and 19, and the incidence rate of smoking is highest between the ages of 20 and 49. The highest consumption is registered among Brazilian women of lower social and economic classes (C, D and E), thus showing that they have less access to information concerning tobacco advertising, or are more strongly influenced by it.^2^

It is common knowledge that there is a causal relationship between cigarette consumption and severe diseases such as brain vascular strokes, acute myocardial infarction, lung cancer, bladder, larynx and pancreatic cancers, and also lung emphysema, among other diseases. In a recent review on the effects of smoking on women's health, Cabar & Carvalho (2003)^2^ reported higher rates of gynecological diseases, particularly vulvovaginitis, sexually transmitted diseases, pelvic inflammation, malignant neoplasm of the uterine cervix and infertility among smokers.

It is furthermore well known that the habit of smoking during pregnancy results in higher rates of spontaneous abortions, ectopic pregnancies, limited fetal growth (dose-dependant occurrence), premature rupture of the membrane and premature birth. In turn, fetuses present higher death rates, lower weights and affected vital signs at birth (as shown by lower Apgar scores).

Over the last two decades, there has been increased public health concern regarding the harm caused to non-smokers who are involuntarily exposed to environmental tobacco smoke.^3^ Studies have reported that such passive smokers have their risk of developing lung cancer increased by 20 to 30 %.^4^ The present study therefore had the aim of assessing the main obstetric and perinatal effects of active, passive or active-passive smoking occurring in the maternity ward of a public hospital in São Paulo.

## PATIENTS AND METHOD

Seven hundred and fifty-eight pregnant patients who were attended in the maternity ward of the Councilor José Storópolli City Hospital in 2001 were randomically selected and after delivery asked to take part in an interview regarding smoking, using a standard questionnaire, before they were discharged from the hospital. The patients were told about the aims of the project and verbally accepted taking part in it. The Ethics Committee of Universidade Federal de São Paulo approved our research project, in accordance with protocol number 681/01. The women taking part in the project consisted of 42 exclusively active smokers, 272 passive smokers, 108 active and simultaneously passive smokers, and 336 non-smokers as the control group.

We considered passive smokers to be those whose spouse, or relative living in the same residence, or co-workers, smoked. Those who had a smoking habit but were not exposed to smoke in their environment were considered to be exclusively active smokers. Those whose smoking habits were associated with close contact with other smokers were considered to be active and passive smokers.

The groups were analyzed according to population characteristics (age group and school education), obstetric characteristics (reports of previous spontaneous abortions, incidence of spontaneous abortions in current pregnancies and incidence of caesarian births) and perinatal characteristics (gestational age, weight and adequacy of weight for gestational age at birth).

To analyze the results obtained, statistical tests were performed. Variance analysis was used to study non-parametric variables and this was complemented by the Tukey method, to measure parametric variables. In all tests performed, the rating was 0.05 or 5% for levels, so as to discard null possibilities. Significant results were marked with an asterisk, and non-significant results were labeled N.S.^5^

## RESULTS

There was a very high rate of smoking during pregnancy (55.7%), which included active smokers (5.5%), passive smokers (35.9%) and active-passive smokers (14.3%). The patients in the groups of active and active-passive smokers were older and had a higher number of pregnancies, in comparison with non-smokers and passive smokers, as seen in Table 1. The active smokers had a lower education level (Table 1).

**Table 1:** Characteristics of the population of women interviewed about their smoking habits during pregnancy

Pregnant women	Non-smokers	Active smokers	Passive smokers	Active/Passive smokers	p
Age (years±)	24.7 (± 6.2)	28.6 (± 6.4)	24.5 (± 6.5)	27.6 (± 6.9)	A, B, C, D*: < 0.01 (*)
Years of education n (%)					
0-7	178 (53.0%)	34 (81.0%)	154 (56.6%)	58 (53.7%)	B, D, E*: < 0.05(*)
8-10	106 (31.5%)	5 (11.9%)	76 (27.9%)	40 (37.0%)	
11 or more	52 (15.5%)	3 (7.1%)	42 (15.5%)	10 (9.3%)	
**Total n (%)**	**336 (100.0%)**	**42 (100.0%)**	**272 (100.0%)**	**108 (100.0%)**	
Parity n(%)					
I	139 (41.4%)	10 (23.8%)	123 (45.2%)	30 (27.8%)	A, B, C, D, E*: < 0.01*
II/IIII	141 (42.0%)	13 (31.0%)	98 (36.0%)	37 (34.2%)	
IV or more	56 (16.6%)	19 (45.2%)	51 (18.8%)	41 (38.0%)	
**Total n (%)**	**336 (100.0%)**	**42 (100.0%)**	**272 (100.0%)**	**108 (100.0%)**	

* *A- Non-smoker ≠ active-passive smoker; B- Non-smoker ≠ active smoker; C- Passive smoker ≠ active + passive smoker; D- Passive smoker ≠ active smoker; E- Active + passive smoker ≠ active smoker*

Although we did not observe a higher rate of spontaneous abortions among current pregnancies, the active smokers presented statistically higher rates of spontaneous abortions for previous pregnancies (Tables 2 and 3). Regarding the delivery, we did not observe a significant difference in rates of caesarian deliveries among the groups analyzed (Table 3).

**Table 2:** Number of previous spontaneous abortions among women interviewed regarding their smoking habits during pregnancy

Pregnant women (B, D, E)*	Non-smokers N (%)	Passive smokers N (%)	Active/passive smokers N (%)	Active smokers N (%)
No abortions	271 (80.7)	225 (82.7%)	83 (76.9%)	21 (50.0%)
One or more abortions	65 (19.3%)	47 (17.3%)	25 (23.1%)	21 (50.0%)
**Total**	**336 (100.0%)**	**272 (100.0%)**	**108 (100.0%)**	**42 (100.0%)**

* *χ^2^ observed = 24.54 (p < 0.01); χ^2^ critical (3 degrees of freedom; 0.05) = 7.81;* * *B- Non-smoker ≠ active smoker; D- passive smoker ≠ active smoker; E- active smoker ≠ active + passive smoker.*

**Table 3:** Obstetric characteristics at the end of the current pregnancy for women interviewed regarding their smoking habits

Obstetric characteristics	Non-smokers N (%)	Passive smokers N (%)	Active/passive smokers N (%)	Active smokers N (%)
Spontaneous abortions	29 (8.8%)	30 (11.2%)	13 (12.6%)	3 (7.3%)
Vaginal Deliveries	233 (70.8%)	180 (67.4%)	75 (72.9%)	30 (73.3)
Caesarian deliveries	67 (20.4%)	57 (21.4%)	15 (14.6%)	8 (19.5%)
TOTAL (*)	**329 (100.0%)**	**267 (100.0%)**	**103 (100.0%)**	**41 (100.0%)**

*Note: patients with ectopic pregnancies (small sample size) and pathological pregnancies terminated before delivery were excluded. χ^2^ observed = 4.09; χ^2^ critical (3 degrees of freedom; 0.05) = 12.59 (non-significant).*

The averages and standard deviations of the gestational age at delivery (in weeks) were 38.7 ± 2.1 for non-smokers, 38.6 ± 2.5 for active smokers, 38.7 ± 2.2 for passive smokers and 38.4 ± 2.4 for active-passive smokers, and there were no statistical differences between these.

The averages and standard deviations of the newborn infants’ weights (in grams) were 3197 ± 496 for non-smokers, 3122 ± 626 for active smokers, 3127 ± 532 for passive smokers and 2961 ± 584 for active-passive smokers. The rate of underweight newborn infants (< 2,500 g) was statistically higher for the groups of smokers (passive and active-passive), in comparison with non-smokers, as seen in Table 4.

**Table 4:** Weights in grams of the newborn infants delivered by women interviewed regarding their smoking habits

Pregnant Women Weight at birth	Non-smokers N %	Active smokers N %	Passive smokers N %	Active/passive smokers N %	Total N %
610-2500 g	14 (4.7)	3 (8.1)	22 (9.4)	15 (16.7)	54 (8.2)
2500-3500 g	204 (68.0)	26 (70.3)	161 (69.1)	64 (71.1)	455 (68.9)
3500-4780 g	82 (27.3)	8 (21.6)	50 (21.5)	11 (12.2)	151 (22.9)
Total*	**300 (100.0)**	**37 (100.0)**	**233 (100.0)**	**90 (100.0)**	**660 (100.00)**

*Note: ectopic and pathological pregnancies terminated before delivery and infants whose birth weight we could not determine were excluded. p < 0.01: non-smokers ≠ active + passive smokers and non-smokers ≠ passive smokers.*

We did not observe any statistically significant differences between the groups regarding adequacy of weight in relation to gestational age.

## DISCUSSION

Our study was performed at a public maternity hospital located in a suburb of the city of São Paulo that attends to patients that are mostly of low economic, social and cultural levels. This may explain the high incidence of women (55.7%) who either voluntarily or involuntarily inhaled cigarette smoke up to the end of their pregnancies. In our interviews, we observed that the active smokers had lower education levels and that only 19.7% (including active smokers and/or passive) had little information about the harm caused by smoking during pregnancy. When they came to reproductive age, approximately 20% of the women in our study smoked (5.5% were exclusively active smokers and 14.3% were active as well as passive smokers). Worse still was the realization that 35.9% of the women who described themselves as non-smokers were actually passive smokers.

The active smokers and the active-passive smokers, who were older and had higher rates of parity than the non-smokers and passive smokers presented longer exposure to the harmful effects of tobacco in the current and previous pregnancies. Although our study presented no increase in the rate of spontaneous abortions for the current pregnancies, active smokers presented statistically higher numbers of previous spontaneous abortions. Contrary to what is seen in the literature, in our study we did not observe a significant difference in the incidence of caesarian deliveries, and premature births or low weight in relation to gestational age at birth. However, the group of smokers (passive and active-passive smokers) showed a higher rate of low-weight infants in comparison with non-smokers.

With regard to smoking rates during pregnancy, the results we reported were similar to those from McLeod et al. (2003),^6^ who reported a frequency of 22.0%. A surprisingly high rate of passive smokers during pregnancy was found in a population from India: 52.0% according to Mathai et al. (1992).^7^ Brodish^8^ reported a spontaneous abortion rate that was two to three times higher for smokers, while Eliopoulos et al. (1996)^9^ reported higher rates of caesarian deliveries, as well as premature births and underweight infants in relation to gestational age at birth. Mainous & Hueston (1994)^10^ reported that the newly born infants of smoking mothers presented twice the risk of being born underweight, as well as all the potential damage that would occur as a result. According to Mathai et al. (1992),^7^ even if the mothers are non-smokers, exposure to environmental smoke during pregnancy could have a deleterious effect on the newly born infant's weight (an average of 55 g less).

Around 4,720 different substances have been identified in cigarette smoke, many of which are pharmacologically active, mutagenic and carcinogenic.^11-13^ Cigarette smoke acts in two ways: centrally and peripherally. Central action occurs when the smoker inhales on the cigarette. This smoke is produced at high temperatures (greater than 950° C) and only pollutes the environment after having been inhaled through the cigarette, filtered through the smoker's lungs and, finally, exhaled. It is the main source of exposure among active smokers. On the other hand, peripheral smoke is produced at lower temperatures (350° C), during the slow spontaneous combustion at the tip of the cigarette, between puffs. This is the type inhaled by passive smokers. It is responsible for 85% of cigarette smoke released directly to the environment. Peripheral smoke differs from the central smoke inhaled by active smokers in that it is not filtered and the nicotine is in a gaseous state.

The typical attitudes among teenagers, coupled with ineffective government policy and lack of restrictions on advertising freely in the media, all contribute to teenagers’ starting to acquire the smoking habit early in our country. The mistaken argument that all the harm to health caused by tobacco is reversible within a short period of time after the smoker quits is the main reason for complacency among teenagers towards their addiction.^8^

Possibly the risk of perinatal and obstetric occurrences is related to the number of cigarettes smoked a day and to the trimester of pregnancy during which highest exposure occurred,^14^ as we know that the fetus gains most weight during the second half of pregnancy.

The influences on the fetus caused by the mother's smoking are a chapter apart regarding the consequences of smoking on health. The fetus is not just any passive smoker inhaling cigarette smoke involuntarily in an open environment; it is a highly vulnerable being whose development is at a stage of risk. When a mother smokes she exposes her fetus not only to the components contained in the cigarette smoke crossing through the placenta, but also to alterations in oxygen rates and placental metabolism and alterations in her own metabolism that are secondary to smoking.

Among the many tobacco components that interfere in pregnancy, we highlight the effects of nicotine and carbon monoxide.^15^ Nicotine acts on the cardiovascular system, causing the release of catecholamines into the mother's circulation, and consequently causing tachycardia, peripheral vasoconstriction and reduction of placental blood flow, resulting in poor nutritional and oxygenation rates for the fetus. Cotinine, a metabolite of nicotine, enhances the vasoconstrictive action of prostaglandin E2 and the accumulation of cotinine in the fetal bloodstream may contribute towards inducing labor prematurely and spontaneous abortion among smokers.^16^

We know that when cigarettes are incompletely burned, there is the production of carbon monoxide, a substance that presents strong affinity towards fetal hemoglobin. Weinberger & Weiss (1996)^17^ reported that a carboxyl-hemoglobin concentration of 10% observed in smokers with severe smoking habits (40 cigarettes a day) resulted in a 60% reduction in fetal blood flow, thereby affecting the transportation of oxygen to and its use in fetal tissue, leading to chronic hypoxia. Thus, carbon monoxide interferes in tissue oxygenation rates in two ways: reducing the blood oxygen transportation capacity and altering the oxy-hemoglobin saturation curve leftwards, thus favoring hypoxemia and resulting in growth restriction.

Smokers appear to present deficiencies in some nutrients such as zinc, carotene and cholesterol. Since all these substances might be inhaled through the nasal mucosa, there is also an effect on the weight of infants born to passive smokers.

Showing women how harmful the effects of passive smoking can be to the weight of newborn infants needs to be taken seriously at routine prenatal examinations, and passive smokers should be identified so as to convey this extremely important information that is often neglected. We emphasize the difficulties in quantifying exposure to cigarettes, because we observed that during the interview active smokers were afraid of being seen to be in the wrong through keeping up their smoking habits throughout pregnancy.

Likewise, it is very difficult to estimate the length of exposure to cigarette smoke inhaled by passive smokers. In 1996, Eliopoulos et al.^9^ suggested measurement of cotinine concentrations in newborn infants’ hair as an effective method for determining the intensity of effects on the organism, especially for passive smokers.

## CONCLUSIONS

As a conclusion, we stress that cigarette smoke, whether or not it is inhaled voluntarily, has a harmful effect towards the mother-infant pair. Because of the importance of healthcare practitioners, who are practically universal within prenatal assistance in all urban areas in Brazil, the pregnancy period should be considered to be the ideal time for stimulating women to quit smoking, given that during this period there is an increase in visits to healthcare practitioners. Likewise, healthcare units and maternity wards should hold lectures and multiprofessional support groups (including doctors, nurses, psychologists and physical therapists) to explain the harm to health and the environment caused by tobacco, and to maintain the interest among both pregnant smokers and their relatives in taking part in various therapeutic methods for quitting smoking.
